# Reduced behavioral withdrawal responses during fear retrieval in adult mice and rats

**DOI:** 10.1177/1744806919876157

**Published:** 2019-09-10

**Authors:** Zhaoxiang Zhou, Kexin Fan, Wantong Shi, Qiyu Chen, Min Zhuo, Jingshan Lu

**Affiliations:** 1 Center for Neuron and Disease, Frontier Institutes of Science and Technology, Xi’an Jiaotong University, Xi’an, China; 2 Department of Physiology, Faculty of Medicine, University of Toronto, Toronto, Ontario, Canada

**Keywords:** Fear, pain threshold, mice, rats

## Abstract

Pain triggers emotional changes in humans and animals, including fear and anxiety. Conversely, fear and anxiety may enhance suffering of patients with pain. However, in animal models of acute pain, it has been reported that fear may inhibit pain by activating endogenous inhibitory systems. In this study, we wanted to examine if behavioral withdrawal responses may be affected during fear retrieval, a condition where fear-associated tone is applied. We found that thermal pain thresholds were significantly increased during fear retrieval. Our results indicate that animals are suffering fear like-events, while their behavioral responses are inhibited. These results indicate that it will be important to evaluate both emotional and behavioral withdrawal responses for future development of new pain medicine.

Pain is an unpleasant sensory experience that triggers centrally related emotional responses such as fear and anxiety.^1,2^ Behavioral withdrawal responses to noxious stimuli are often used to investigate pain mechanisms. Inhibition of these withdrawal responses is assumed to be analgesic. However, many new drugs discovered in animal pain models have failed in clinical studies. One possible explanation is that animal behavioral tests may not be appropriate or not sufficient to evaluate pain perception in animals.^3^ It is known that cortical areas such as the anterior cingulate cortex (ACC) and insular cortex are critical for pain perception.^2^ It becomes clear that understanding sensory synaptic modulation at both spinal and cortical levels is important for designing better treatment for chronic pain. Purely evaluating behavioral withdrawal responses may not be sufficient to understand pain perception.

Fear and anxiety are two common emotional responses to chronic pain. It has been reported that anxiety and possibly fear may enhance the suffering of patients with chronic pain.^4,5^ In animal models of anxiety, it has been reported that pain can be enhanced.^6^ Cortical regions that are reported in pain perception are also indicated in fear memory.^7^ Furthermore, ACC circuits have also been indicated in fear retrieval.^8^ We are interested in investigating if animal behavioral responses will be facilitated or enhanced during the retrieval of fear, considering that many ACC neurons will be activated.^9^ Animal models have been used to mimic human pain, since animals cannot communicate with verbal reports.^10^ However, it may not be appropriate to use animal withdrawal responses to estimate the actual pain responses in animals. Animals may experience unpleasant fear, while behavioral responses may be inhibited.^11–13^

In this study, we used a model of tone-cued fear conditioning in mice and rats. All procedures and handling of animals were performed with permission according to the guidelines of Xi’an Jiaotong University. Fear conditioning was performed in an isolated shock chamber. After 3 min of habituation, animals received the tone/shock pairing (a 30 s tone and a 2 s shock starting at 28 s; three shock/tone pairings were delivered at 30 s intervals). Based on the model or control groups (Figure 1(a)), the withdrawal latency to noxious radiant heat was measured in mice and rats, using the “plantar test” apparatus with or without a tone. To measure latency, a cylindrical pipe with an infrared emitter is situated under the glass floor. It records latency when the animal moves its feet.

**Figure 1.**
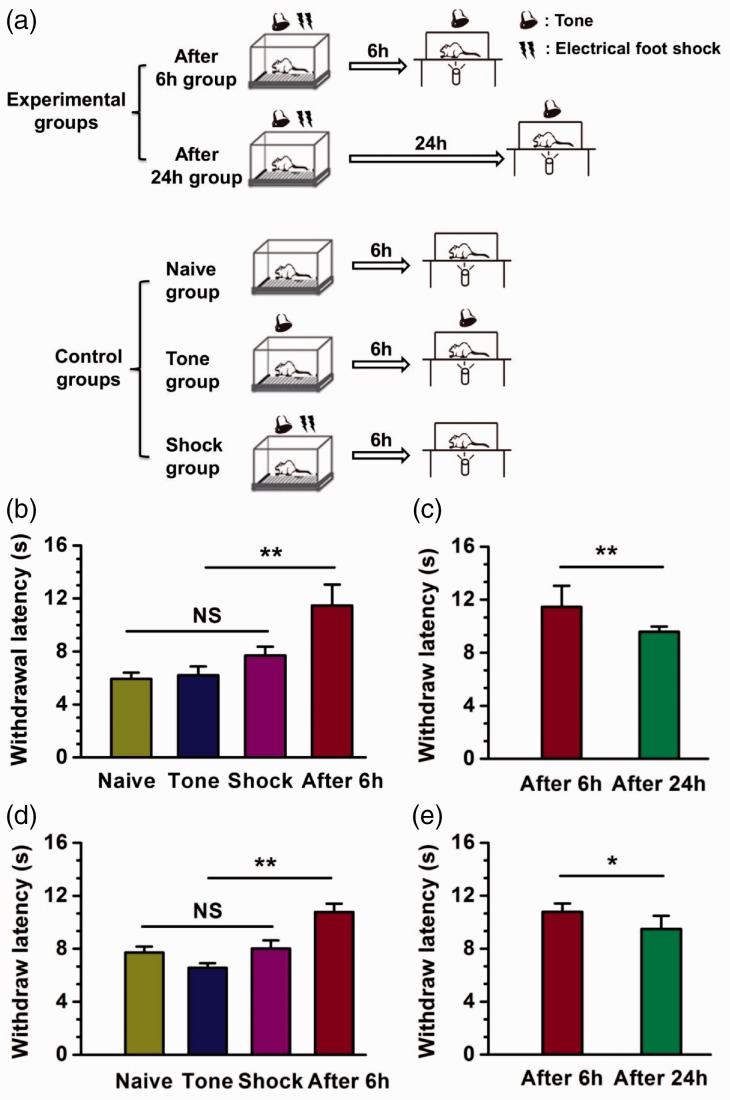
Reduced behavioral withdrawal responses to noxious heat during fear retrieval in mice and rats. (a) Schematic diagram showing the different fear conditioning models preformed on five rodent groups. Experiment group rodents were exposed to a context with tone/shock pairing. The tone was 96 dB with 2.2 kHz for 30 s. The foot shock (0.75 mA for mice, 2.0 mA for rats) lasted for 2 s. The withdrawal latency to radiant heat was measured after foot shock after 6 h or 24 h with tone pairing. For naive group, rodents were just exposed to a context, and the withdrawal latency was measured without a tone after 6 h. For tone group, rodents were exposed to a context with a tone alone, and the withdrawal latency was measured with a tone after 6 h. For shock group, rodents were exposed to a context with tone/shock pairing, and the withdrawal latency was measured without a tone after 6 h. (b) The withdrawal latency was increased significantly in after 6-h group compared with control groups in mice. n = 12 for after 6-h group (eight male and four female mice), n = 8 for each control group (four male and four female mice), **p < 0.01, N.S. not significant. There was no difference in control groups. (c) The withdrawal latency was decreased in after 24-h group compared with 6 h after fear conditioning in mice. n = 12 for after 24-h groups (eight male and four female mice), **p < 0.01, N.S. not significant. The values of after 6-h group in (c) are totally the same with (b). (d) The withdrawal latency was increased significantly in after 6-h group compared with control groups in rats. n = 12 for after 6-h group (eight male and four female rats), n = 8 for each control group (four male and four female rats), **p < 0.01, N.S. not significant. There was no difference in control groups. (e) The withdrawal latency was decreased in after 24-h group compared with 6-h after fear conditioning in rats. n = 8 for after 24-h group (four male and four female mice), *p < 0.05, N.S. not significant. The values of after 6-h group in (e) are totally the same with (d).

We first examined the contribution of fear conditioning to the thermal pain threshold in mice. Considering the behavioral sex difference in pain research, experiments were applied in both male and female mice. No significant differences between male and female mice were found, and thus data are pooled together. There was no significant difference in latency of thermal pain response after training among naive group animals, tone group animals, and shock group animals (Figure 1(b), naive group: 7.71 ± 0.46 s, tone group: 6.57 ± 0.33 s, shock group: 8.02 ± 0.60 s, n = 8, male: 4, female: 4). Compared to the naive, tone, and shock groups, mice in fear conditioning showed increased latency 6 h after the tone-cued fear conditioning (Figure 1(b), naive group: 10.78 ± 0.63 s, **p <* *0.01, n = 12, male: 8, female: 4). In addition, we also measured the paw withdrawal latency 24 h after tone-cued fear conditioning (Figure 1(c), 9.49 ± 0.99 s, *p <* *0.05, n = 12, male: 8, female: 4) and compared them with 6 h. We found that pain threshold was reduced with less retrieval of fear.

To examine if similar results can be found in adult rats, we repeated the experiments in male and female adult rats. Consistent with our experiments in mice, there was also no significant difference among naive group animals, tone group animals, and shock group animals (Figure 1(d), naive group: 5.93 ± 0.46 s, tone group: 6.20 ± 0.66 s, shock group: 7.69 ± 0.67 s, n = 8, male: 4, female: 4). Retrieval of fear also increased withdrawal latency in rats 6 h after the tone-cued fear conditioning (Figure 1(d), 11.47 ± 1.58 s, **p <* *0.01, n = 12, male: 8, female: 4). The withdrawal latency at 24 h after fear conditioning was still significantly decreased (Figure 1(e), 9.59 ± 0.39 s, **p <* *0.01, n = 8, male: 4, female: 4). We did not find significant difference in these results collected from male and female rats; thus, data were pooled together. In control groups, neither tone nor shock alone altered the withdrawal latency. However, the retrieval group (after 6 h group) that had received shock with tone during pain test showed prolonged thermal latency.

In conclusion, by applying the tone-cued fear conditioning, we discovered that behavioral withdrawal responses to noxious heat were significantly reduced during the retrieval of fear in adult mice and rats. It is known that ACC neurons are activated during fear conditioning and retrieval.^8,13^ Our recent study demonstrated that ACC neurons exert a descending facilitative effect on spinal nociceptive transmission.^9^ Thus, we predict that behavioral withdrawal responses to noxious stimuli may be facilitated during fear retrieval. However, in this study, we found that behavioral withdrawal responses to noxious stimuli were significantly reduced during fear retrieval. It is possible that such inhibition is due to the activation of endogenous descending inhibitory systems. It is known that periaqueductal gray (PAG) neurons are activated during fear or fear retrieval.^14,15^ PAG activates descending inhibitory systems and inhibit spinal nociceptive transmission.^15,16^ In addition, the amygdala, a key region for fear memory,^17,18^ may also be involved. Activation of amygdala neurons during fear retrieval may affect spinal cord pain transmission by triggering PAG-related descending inhibitory pathway.^19^ Future studies are clearly needed to map the neuronal circuit that inhibits spinal nociceptive responses during fear. Our present study also indicates that it is incomplete to evaluate emotional pain by measuring behavioral withdrawal responses only. It is also important to determine the emotional fear or anxiety status while evaluating spinal nociceptive or behavioral responses.
